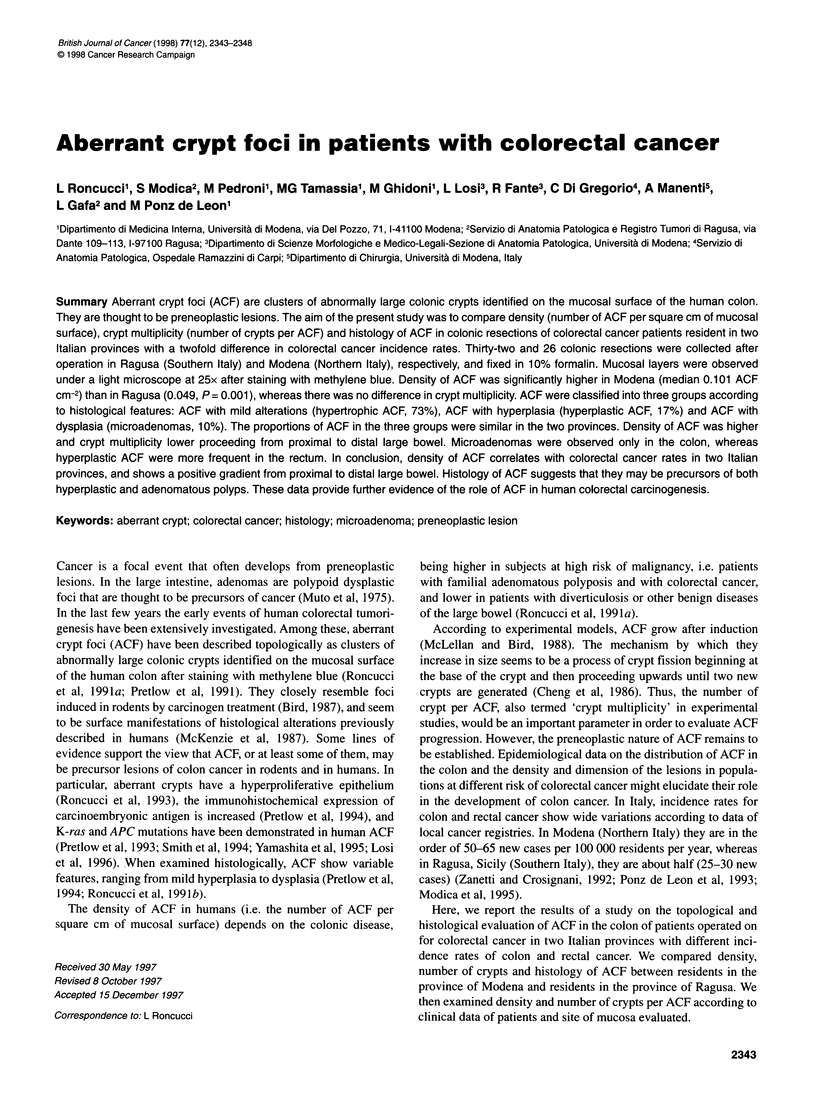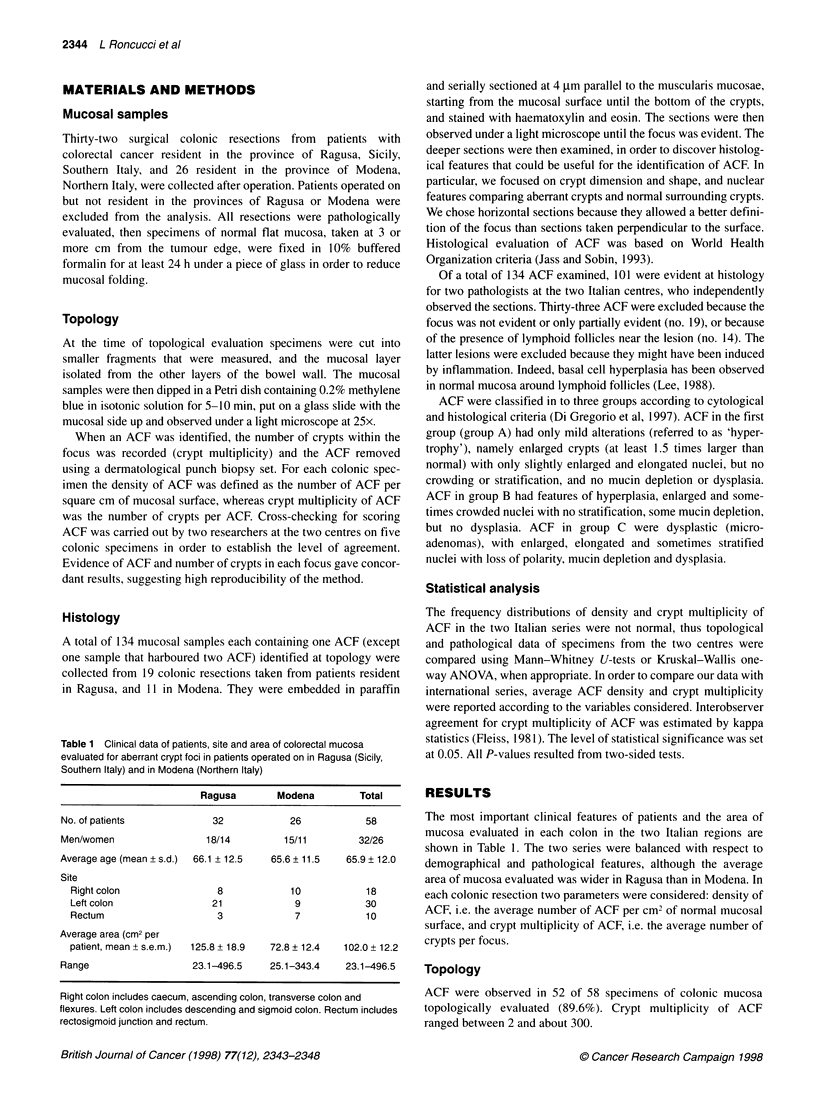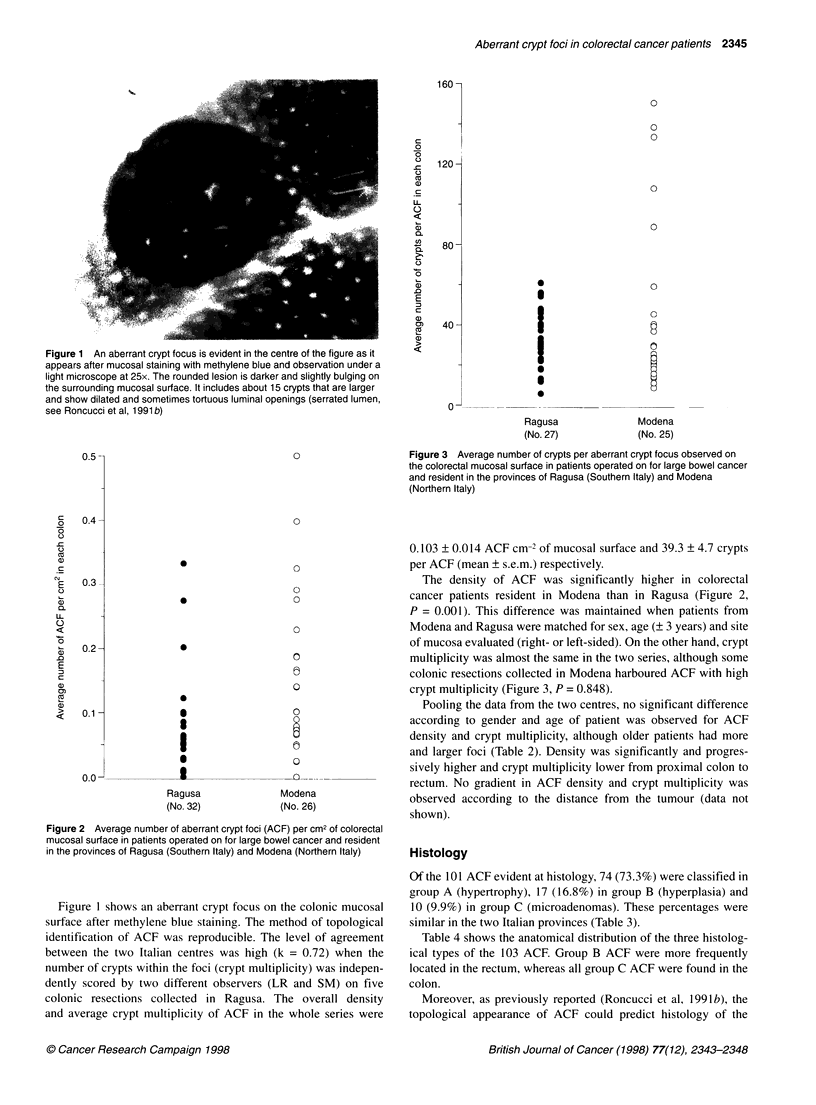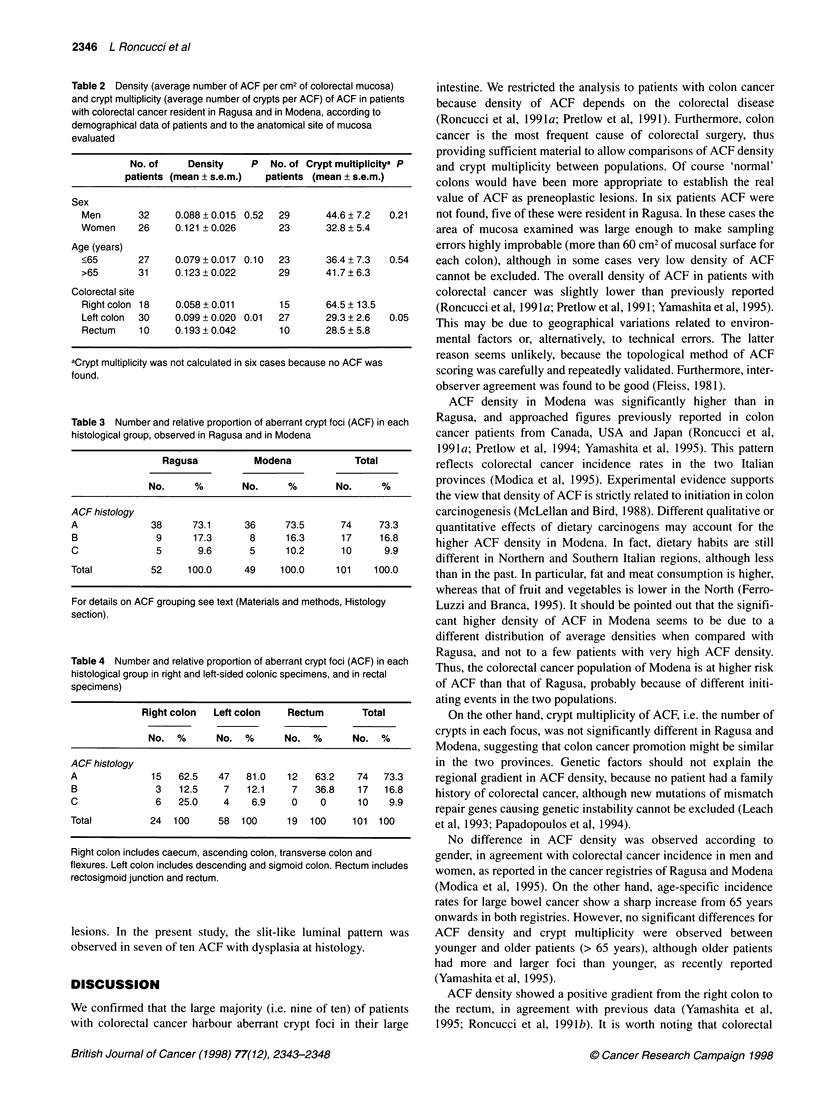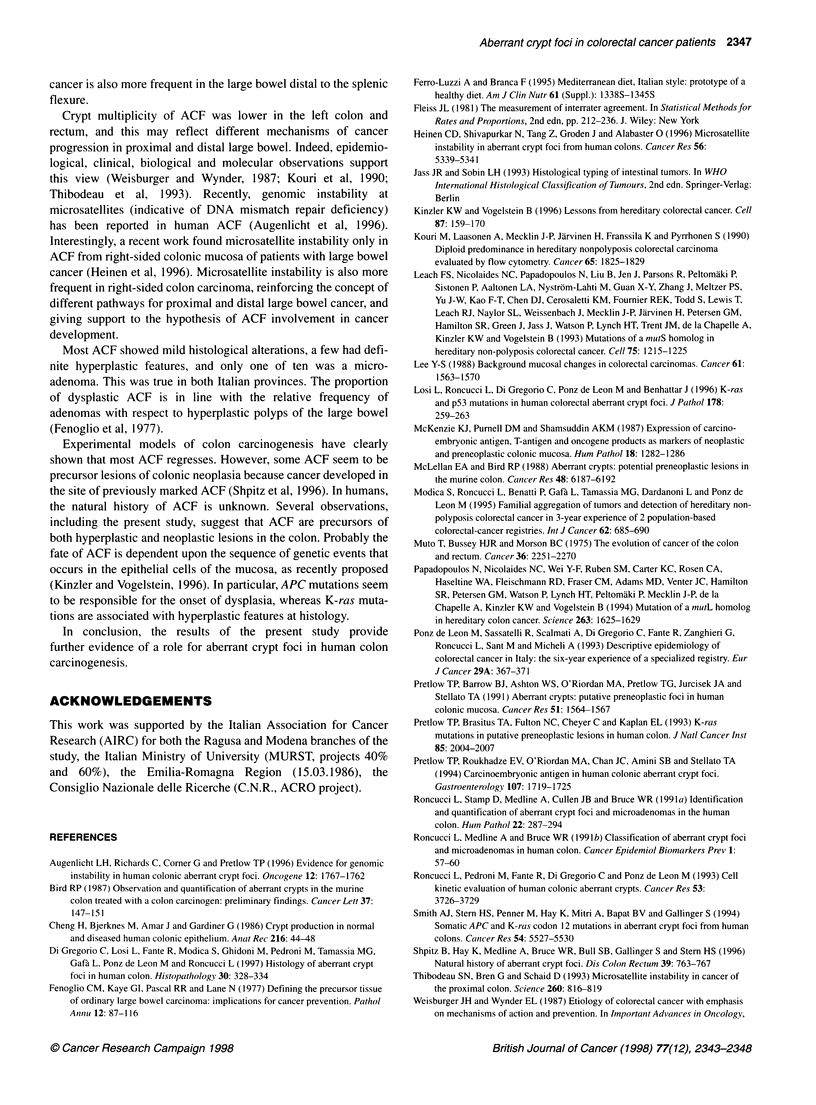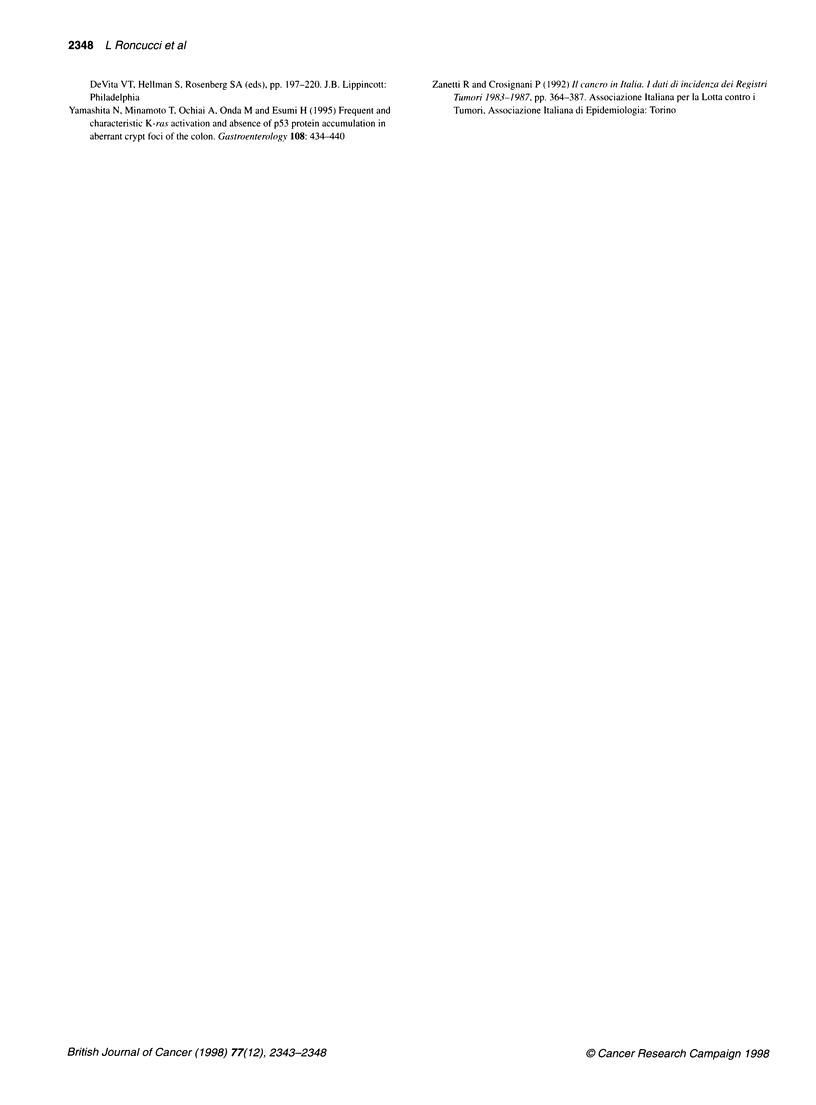# Aberrant crypt foci in patients with colorectal cancer.

**DOI:** 10.1038/bjc.1998.389

**Published:** 1998-06

**Authors:** L. Roncucci, S. Modica, M. Pedroni, M. G. Tamassia, M. Ghidoni, L. Losi, R. Fante, C. Di Gregorio, A. Manenti, L. Gafa, M. Ponz de Leon

**Affiliations:** Dipartimento di Medicina Interna, UniversitÃ di Modena, Italy.

## Abstract

**Images:**


British Journal of Cancer (1998) 77(12), 2343–2348
© 1998 Cancer Research Campaign

### Aberrant crypt foci in patients with colorectal cancer

**L Roncucci[1], S Modica[2], M Pedroni[1], MG Tamassia[1], M Ghidoni[1], L Losi[3], R Fante[3], C Di Gregorio[4], A Manenti[5], L Gafa[2] and M Ponz de Leon[1]**

[1]Dipartimento di Medicina Interna, Università di Modena, via Del Pozzo, 71, I-41100 Modena; [2]Servizio di Anatomia Patologica e Registro Tumori di Ragusa, via Dante 109–113, I-97100 Ragusa; [3]Dipartimento di Scienze Morfologiche e Medico-Legali-Sezione di Anatomia Patologica, Università di Modena; [4]Servizio di Anatomia Patologica, Ospedale Ramazzini di Carpi; [5]Dipartimento di Chirurgia, Università di Modena, Italy

**Summary** Aberrant crypt foci (ACF) are clusters of abnormally large colonic crypts identified on the mucosal surface of the human colon. They are thought to be preneoplastic lesions. The aim of the present study was to compare density (number of ACF per square cm of mucosal surface), crypt multiplicity (number of crypts per ACF) and histology of ACF in colonic resections of colorectal cancer patients resident in two Italian provinces with a twofold difference in colorectal cancer incidence rates. Thirty-two and 26 colonic resections were collected after operation in Ragusa (Southern Italy) and Modena (Northern Italy), respectively, and fixed in 10% formalin. Mucosal layers were observed under a light microscope at 25× after staining with methylene blue. Density of ACF was significantly higher in Modena (median 0.101 ACF $cm^{-2}$) than in Ragusa (0.049, $P = 0.001$), whereas there was no difference in crypt multiplicity. ACF were classified into three groups according to histological features: ACF with mild alterations (hypertrophic ACF, 73%), ACF with hyperplasia (hyperplastic ACF, 17%) and ACF with dysplasia (microadenomas, 10%). The proportions of ACF in the three groups were similar in the two provinces. Density of ACF was higher and crypt multiplicity lower proceeding from proximal to distal large bowel. Microadenomas were observed only in the colon, whereas hyperplastic ACF were more frequent in the rectum. In conclusion, density of ACF correlates with colorectal cancer rates in two Italian provinces, and shows a positive gradient from proximal to distal large bowel. Histology of ACF suggests that they may be precursors of both hyperplastic and adenomatous polyps. These data provide further evidence of the role of ACF in human colorectal carcinogenesis.

**Keywords:** aberrant crypt; colorectal cancer; histology; microadenoma; preneoplastic lesion

Cancer is a focal event that often develops from preneoplastic lesions. In the large intestine, adenomas are polypoid dysplastic foci that are thought to be precursors of cancer (Muto et al, 1975). In the last few years the early events of human colorectal tumorigenesis have been extensively investigated. Among these, aberrant crypt foci (ACF) have been described topologically as clusters of abnormally large colonic crypts identified on the mucosal surface of the human colon after staining with methylene blue (Roncucci et al, 1991*a*; Pretlow et al, 1991). They closely resemble foci induced in rodents by carcinogen treatment (Bird, 1987), and seem to be surface manifestations of histological alterations previously described in humans (McKenzie et al, 1987). Some lines of evidence support the view that ACF, or at least some of them, may be precursor lesions of colon cancer in rodents and in humans. In particular, aberrant crypts have a hyperproliferative epithelium (Roncucci et al, 1993), the immunohistochemical expression of carcinoembryonic antigen is increased (Pretlow et al, 1994), and K-*ras* and *APC* mutations have been demonstrated in human ACF (Pretlow et al, 1993; Smith et al, 1994; Yamashita et al, 1995; Losi et al, 1996). When examined histologically, ACF show variable features, ranging from mild hyperplasia to dysplasia (Pretlow et al, 1994; Roncucci et al, 1991*b*).

The density of ACF in humans (i.e. the number of ACF per square cm of mucosal surface) depends on the colonic disease, being higher in subjects at high risk of malignancy, i.e. patients with familial adenomatous polyposis and with colorectal cancer, and lower in patients with diverticulosis or other benign diseases of the large bowel (Roncucci et al, 1991*a*).

According to experimental models, ACF grow after induction (McLellan and Bird, 1988). The mechanism by which they increase in size seems to be a process of crypt fission beginning at the base of the crypt and then proceeding upwards until two new crypts are generated (Cheng et al, 1986). Thus, the number of crypt per ACF, also termed 'crypt multiplicity' in experimental studies, would be an important parameter in order to evaluate ACF progression. However, the preneoplastic nature of ACF remains to be established. Epidemiological data on the distribution of ACF in the colon and the density and dimension of the lesions in populations at different risk of colorectal cancer might elucidate their role in the development of colon cancer. In Italy, incidence rates for colon and rectal cancer show wide variations according to data of local cancer registries. In Modena (Northern Italy) they are in the order of 50–65 new cases per 100 000 residents per year, whereas in Ragusa, Sicily (Southern Italy), they are about half (25–30 new cases) (Zanetti and Crosignani, 1992; Ponz de Leon et al, 1993; Modica et al, 1995).

Here, we report the results of a study on the topological and histological evaluation of ACF in the colon of patients operated on for colorectal cancer in two Italian provinces with different incidence rates of colon and rectal cancer. We compared density, number of crypts and histology of ACF between residents in the province of Modena and residents in the province of Ragusa. We then examined density and number of crypts per ACF according to clinical data of patients and site of mucosa evaluated.

*Received 30 May 1997*
*Revised 8 October 1997*
*Accepted 15 December 1997*

*Correspondence to:* L Roncucci

### MATERIALS AND METHODS

### Mucosal samples

Thirty-two surgical colonic resections from patients with colorectal cancer resident in the province of Ragusa, Sicily, Southern Italy, and 26 resident in the province of Modena, Northern Italy, were collected after operation. Patients operated on but not resident in the provinces of Ragusa or Modena were excluded from the analysis. All resections were pathologically evaluated, then specimens of normal flat mucosa, taken at 3 or more cm from the tumour edge, were fixed in 10% buffered formalin for at least 24 h under a piece of glass in order to reduce mucosal folding.

### Topology

At the time of topological evaluation specimens were cut into smaller fragments that were measured, and the mucosal layer isolated from the other layers of the bowel wall. The mucosal samples were then dipped in a Petri dish containing 0.2% methylene blue in isotonic solution for 5–10 min, put on a glass slide with the mucosal side up and observed under a light microscope at 25×.

When an ACF was identified, the number of crypts within the focus was recorded (crypt multiplicity) and the ACF removed using a dermatological punch biopsy set. For each colonic specimen the density of ACF was defined as the number of ACF per square cm of mucosal surface, whereas crypt multiplicity of ACF was the number of crypts per ACF. Cross-checking for scoring ACF was carried out by two researchers at the two centres on five colonic specimens in order to establish the level of agreement. Evidence of ACF and number of crypts in each focus gave concordant results, suggesting high reproducibility of the method.

### Histology

A total of 134 mucosal samples each containing one ACF (except one sample that harboured two ACF) identified at topology were collected from 19 colonic resections taken from patients resident in Ragusa, and 11 in Modena. They were embedded in paraffin and serially sectioned at 4 μm parallel to the muscularis mucosae, starting from the mucosal surface until the bottom of the crypts, and stained with haematoxylin and eosin. The sections were then observed under a light microscope until the focus was evident. The deeper sections were then examined, in order to discover histological features that could be useful for the identification of ACF. In particular, we focused on crypt dimension and shape, and nuclear features comparing aberrant crypts and normal surrounding crypts. We chose horizontal sections because they allowed a better definition of the focus than sections taken perpendicular to the surface. Histological evaluation of ACF was based on World Health Organization criteria (Jass and Sobin, 1993).

Of a total of 134 ACF examined, 101 were evident at histology for two pathologists at the two Italian centres, who independently observed the sections. Thirty-three ACF were excluded because the focus was not evident or only partially evident (no. 19), or because of the presence of lymphoid follicles near the lesion (no. 14). The latter lesions were excluded because they might have been induced by inflammation. Indeed, basal cell hyperplasia has been observed in normal mucosa around lymphoid follicles (Lee, 1988).

ACF were classified in to three groups according to cytological and histological criteria (Di Gregorio et al, 1997). ACF in the first group (group A) had only mild alterations (referred to as 'hypertrophy'), namely enlarged crypts (at least 1.5 times larger than normal) with only slightly enlarged and elongated nuclei, but no crowding or stratification, and no mucin depletion or dysplasia. ACF in group B had features of hyperplasia, enlarged and sometimes crowded nuclei with no stratification, some mucin depletion, but no dysplasia. ACF in group C were dysplastic (microadenomas), with enlarged, elongated and sometimes stratified nuclei with loss of polarity, mucin depletion and dysplasia.

**Table 1** Clinical data of patients, site and area of colorectal mucosa evaluated for aberrant crypt foci in patients operated on in Ragusa (Sicily, Southern Italy) and in Modena (Northern Italy)

| | Ragusa | Modena | Total |
|---|---|---|---|
| No. of patients | 32 | 26 | 58 |
| Men/women | 18/14 | 15/11 | 32/26 |
| Average age (mean ± s.d.) | 66.1 ± 12.5 | 65.6 ± 11.5 | 65.9 ± 12.0 |
| Site | | | |
| Right colon | 8 | 10 | 18 |
| Left colon | 21 | 9 | 30 |
| Rectum | 3 | 7 | 10 |
| Average area ($cm^2$ per patient, mean ± s.e.m.) | 125.8 ± 18.9 | 72.8 ± 12.4 | 102.0 ± 12.2 |
| Range | 23.1–496.5 | 25.1–343.4 | 23.1–496.5 |

Right colon includes caecum, ascending colon, transverse colon and flexures. Left colon includes descending and sigmoid colon. Rectum includes rectosigmoid junction and rectum.

### Statistical analysis

The frequency distributions of density and crypt multiplicity of ACF in the two Italian series were not normal, thus topological and pathological data of specimens from the two centres were compared using Mann–Whitney $U$-tests or Kruskal–Wallis one-way ANOVA, when appropriate. In order to compare our data with international series, average ACF density and crypt multiplicity were reported according to the variables considered. Interobserver agreement for crypt multiplicity of ACF was estimated by kappa statistics (Fleiss, 1981). The level of statistical significance was set at 0.05. All $P$-values resulted from two-sided tests.

### RESULTS

The most important clinical features of patients and the area of mucosa evaluated in each colon in the two Italian regions are shown in Table 1. The two series were balanced with respect to demographical and pathological features, although the average area of mucosa evaluated was wider in Ragusa than in Modena. In each colonic resection two parameters were considered: density of ACF, i.e. the average number of ACF per $cm^2$ of normal mucosal surface, and crypt multiplicity of ACF, i.e. the average number of crypts per focus.

### Topology

ACF were observed in 52 of 58 specimens of colonic mucosa topologically evaluated (89.6%). Crypt multiplicity of ACF ranged between 2 and about 300.

© Cancer Research Campaign 1998

**Figure 1** An aberrant crypt focus is evident in the centre of the figure as it appears after mucosal staining with methylene blue and observation under a light microscope at 25×. The rounded lesion is darker and slightly bulging on the surrounding mucosal surface. It includes about 15 crypts that are larger and show dilated and sometimes tortuous luminal openings (serrated lumen, see Roncucci et al, 1991*b*)

**Figure 2** Average number of aberrant crypt foci (ACF) per cm$^2$ of colorectal mucosal surface in patients operated on for large bowel cancer and resident in the provinces of Ragusa (Southern Italy) and Modena (Northern Italy)

**Figure 3** Average number of crypts per aberrant crypt focus observed on the colorectal mucosal surface in patients operated on for large bowel cancer and resident in the provinces of Ragusa (Southern Italy) and Modena (Northern Italy)

Figure 1 shows an aberrant crypt focus on the colonic mucosal surface after methylene blue staining. The method of topological identification of ACF was reproducible. The level of agreement between the two Italian centres was high (k = 0.72) when the number of crypts within the foci (crypt multiplicity) was independently scored by two different observers (LR and SM) on five colonic resections collected in Ragusa. The overall density and average crypt multiplicity of ACF in the whole series were 0.103 ± 0.014 ACF cm$^{-2}$ of mucosal surface and 39.3 ± 4.7 crypts per ACF (mean ± s.e.m.) respectively.

The density of ACF was significantly higher in colorectal cancer patients resident in Modena than in Ragusa (Figure 2, $P = 0.001$). This difference was maintained when patients from Modena and Ragusa were matched for sex, age (± 3 years) and site of mucosa evaluated (right- or left-sided). On the other hand, crypt multiplicity was almost the same in the two series, although some colonic resections collected in Modena harboured ACF with high crypt multiplicity (Figure 3, $P = 0.848$).

Pooling the data from the two centres, no significant difference according to gender and age of patient was observed for ACF density and crypt multiplicity, although older patients had more and larger foci (Table 2). Density was significantly and progressively higher and crypt multiplicity lower from proximal colon to rectum. No gradient in ACF density and crypt multiplicity was observed according to the distance from the tumour (data not shown).

### Histology

Of the 101 ACF evident at histology, 74 (73.3%) were classified in group A (hypertrophy), 17 (16.8%) in group B (hyperplasia) and 10 (9.9%) in group C (microadenomas). These percentages were similar in the two Italian provinces (Table 3).

Table 4 shows the anatomical distribution of the three histological types of the 103 ACF. Group B ACF were more frequently located in the rectum, whereas all group C ACF were found in the colon.

Moreover, as previously reported (Roncucci et al, 1991*b*), the topological appearance of ACF could predict histology of the

© Cancer Research Campaign 1998

**Table 2** Density (average number of ACF per cm² of colorectal mucosa) and crypt multiplicity (average number of crypts per ACF) of ACF in patients with colorectal cancer resident in Ragusa and in Modena, according to demographical data of patients and to the anatomical site of mucosa evaluated

| | No. of patients | Density (mean ± s.e.m.) | P | No. of patients | Crypt multiplicity[a] (mean ± s.e.m.) | P |
|---|---|---|---|---|---|---|
| Sex | | | | | | |
| Men | 32 | 0.088 ± 0.015 | 0.52 | 29 | 44.6 ± 7.2 | 0.21 |
| Women | 26 | 0.121 ± 0.026 | | 23 | 32.8 ± 5.4 | |
| Age (years) | | | | | | |
| ≤65 | 27 | 0.079 ± 0.017 | 0.10 | 23 | 36.4 ± 7.3 | 0.54 |
| >65 | 31 | 0.123 ± 0.022 | | 29 | 41.7 ± 6.3 | |
| Colorectal site | | | | | | |
| Right colon | 18 | 0.058 ± 0.011 | | 15 | 64.5 ± 13.5 | |
| Left colon | 30 | 0.099 ± 0.020 | 0.01 | 27 | 29.3 ± 2.6 | 0.05 |
| Rectum | 10 | 0.193 ± 0.042 | | 10 | 28.5 ± 5.8 | |

[a]Crypt multiplicity was not calculated in six cases because no ACF was found.

**Table 3** Number and relative proportion of aberrant crypt foci (ACF) in each histological group, observed in Ragusa and in Modena

| | Ragusa | | Modena | | Total | |
|---|---|---|---|---|---|---|
| | No. | % | No. | % | No. | % |
| *ACF histology* | | | | | | |
| A | 38 | 73.1 | 36 | 73.5 | 74 | 73.3 |
| B | 9 | 17.3 | 8 | 16.3 | 17 | 16.8 |
| C | 5 | 9.6 | 5 | 10.2 | 10 | 9.9 |
| Total | 52 | 100.0 | 49 | 100.0 | 101 | 100.0 |

For details on ACF grouping see text (Materials and methods, Histology section).

**Table 4** Number and relative proportion of aberrant crypt foci (ACF) in each histological group in right and left-sided colonic specimens, and in rectal specimens)

| | Right colon | | Left colon | | Rectum | | Total | |
|---|---|---|---|---|---|---|---|---|
| | No. | % | No. | % | No. | % | No. | % |
| *ACF histology* | | | | | | | | |
| A | 15 | 62.5 | 47 | 81.0 | 12 | 63.2 | 74 | 73.3 |
| B | 3 | 12.5 | 7 | 12.1 | 7 | 36.8 | 17 | 16.8 |
| C | 6 | 25.0 | 4 | 6.9 | 0 | 0 | 10 | 9.9 |
| Total | 24 | 100 | 58 | 100 | 19 | 100 | 101 | 100 |

Right colon includes caecum, ascending colon, transverse colon and flexures. Left colon includes descending and sigmoid colon. Rectum includes rectosigmoid junction and rectum.

lesions. In the present study, the slit-like luminal pattern was observed in seven of ten ACF with dysplasia at histology.

### DISCUSSION

We confirmed that the large majority (i.e. nine of ten) of patients with colorectal cancer harbour aberrant crypt foci in their large intestine. We restricted the analysis to patients with colon cancer because density of ACF depends on the colorectal disease (Roncucci et al, 1991*a*; Pretlow et al, 1991). Furthermore, colon cancer is the most frequent cause of colorectal surgery, thus providing sufficient material to allow comparisons of ACF density and crypt multiplicity between populations. Of course 'normal' colons would have been more appropriate to establish the real value of ACF as preneoplastic lesions. In six patients ACF were not found, five of these were resident in Ragusa. In these cases the area of mucosa examined was large enough to make sampling errors highly improbable (more than 60 cm² of mucosal surface for each colon), although in some cases very low density of ACF cannot be excluded. The overall density of ACF in patients with colorectal cancer was slightly lower than previously reported (Roncucci et al, 1991*a*; Pretlow et al, 1991; Yamashita et al, 1995). This may be due to geographical variations related to environmental factors or, alternatively, to technical errors. The latter reason seems unlikely, because the topological method of ACF scoring was carefully and repeatedly validated. Furthermore, inter-observer agreement was found to be good (Fleiss, 1981).

ACF density in Modena was significantly higher than in Ragusa, and approached figures previously reported in colon cancer patients from Canada, USA and Japan (Roncucci et al, 1991*a*; Pretlow et al, 1994; Yamashita et al, 1995). This pattern reflects colorectal cancer incidence rates in the two Italian provinces (Modica et al, 1995). Experimental evidence supports the view that density of ACF is strictly related to initiation in colon carcinogenesis (McLellan and Bird, 1988). Different qualitative or quantitative effects of dietary carcinogens may account for the higher ACF density in Modena. In fact, dietary habits are still different in Northern and Southern Italian regions, although less than in the past. In particular, fat and meat consumption is higher, whereas that of fruit and vegetables is lower in the North (Ferro-Luzzi and Branca, 1995). It should be pointed out that the significant higher density of ACF in Modena seems to be due to a different distribution of average densities when compared with Ragusa, and not to a few patients with very high ACF density. Thus, the colorectal cancer population of Modena is at higher risk of ACF than that of Ragusa, probably because of different initiating events in the two populations.

On the other hand, crypt multiplicity of ACF, i.e. the number of crypts in each focus, was not significantly different in Ragusa and Modena, suggesting that colon cancer promotion might be similar in the two provinces. Genetic factors should not explain the regional gradient in ACF density, because no patient had a family history of colorectal cancer, although new mutations of mismatch repair genes causing genetic instability cannot be excluded (Leach et al, 1993; Papadopoulos et al, 1994).

No difference in ACF density was observed according to gender, in agreement with colorectal cancer incidence in men and women, as reported in the cancer registries of Ragusa and Modena (Modica et al, 1995). On the other hand, age-specific incidence rates for large bowel cancer show a sharp increase from 65 years onwards in both registries. However, no significant differences for ACF density and crypt multiplicity were observed between younger and older patients (> 65 years), although older patients had more and larger foci than younger, as recently reported (Yamashita et al, 1995).

ACF density showed a positive gradient from the right colon to the rectum, in agreement with previous data (Yamashita et al, 1995; Roncucci et al, 1991*b*). It is worth noting that colorectal

© Cancer Research Campaign 1998

cancer is also more frequent in the large bowel distal to the splenic flexure.

Crypt multiplicity of ACF was lower in the left colon and rectum, and this may reflect different mechanisms of cancer progression in proximal and distal large bowel. Indeed, epidemiological, clinical, biological and molecular observations support this view (Weisburger and Wynder, 1987; Kouri et al, 1990; Thibodeau et al, 1993). Recently, genomic instability at microsatellites (indicative of DNA mismatch repair deficiency) has been reported in human ACF (Augenlicht et al, 1996). Interestingly, a recent work found microsatellite instability only in ACF from right-sided colonic mucosa of patients with large bowel cancer (Heinen et al, 1996). Microsatellite instability is also more frequent in right-sided colon carcinoma, reinforcing the concept of different pathways for proximal and distal large bowel cancer, and giving support to the hypothesis of ACF involvement in cancer development.

Most ACF showed mild histological alterations, a few had definite hyperplastic features, and only one of ten was a microadenoma. This was true in both Italian provinces. The proportion of dysplastic ACF is in line with the relative frequency of adenomas with respect to hyperplastic polyps of the large bowel (Fenoglio et al, 1977).

Experimental models of colon carcinogenesis have clearly shown that most ACF regresses. However, some ACF seem to be precursor lesions of colonic neoplasia because cancer developed in the site of previously marked ACF (Shpitz et al, 1996). In humans, the natural history of ACF is unknown. Several observations, including the present study, suggest that ACF are precursors of both hyperplastic and neoplastic lesions in the colon. Probably the fate of ACF is dependent upon the sequence of genetic events that occurs in the epithelial cells of the mucosa, as recently proposed (Kinzler and Vogelstein, 1996). In particular, *APC* mutations seem to be responsible for the onset of dysplasia, whereas K-*ras* mutations are associated with hyperplastic features at histology.

In conclusion, the results of the present study provide further evidence of a role for aberrant crypt foci in human colon carcinogenesis.

### ACKNOWLEDGEMENTS

This work was supported by the Italian Association for Cancer Research (AIRC) for both the Ragusa and Modena branches of the study, the Italian Ministry of University (MURST, projects 40% and 60%), the Emilia-Romagna Region (15.03.1986), the Consiglio Nazionale delle Ricerche (C.N.R., ACRO project).

### REFERENCES

Augenlicht LH, Richards C, Corner G and Pretlow TP (1996) Evidence for genomic instability in human colonic aberrant crypt foci. *Oncogene* **12**: 1767–1762

Bird RP (1987) Observation and quantification of aberrant crypts in the murine colon treated with a colon carcinogen: preliminary findings. *Cancer Lett* **37**: 147–151

Cheng H, Bjerknes M, Amar J and Gardiner G (1986) Crypt production in normal and diseased human colonic epithelium. *Anat Rec* **216**: 44–48

Di Gregorio C, Losi L, Fante R, Modica S, Ghidoni M, Pedroni M, Tamassia MG, Gafà L, Ponz de Leon M and Roncucci L (1997) Histology of aberrant crypt foci in human colon. *Histopathology* **30**: 328–334

Fenoglio CM, Kaye GI, Pascal RR and Lane N (1977) Defining the precursor tissue of ordinary large bowel carcinoma: implications for cancer prevention. *Pathol Annu* **12**: 87–116

Ferro-Luzzi A and Branca F (1995) Mediterranean diet, Italian style: prototype of a healthy diet. *Am J Clin Nutr* **61** (Suppl.): 1338S–1345S

Fleiss JL (1981) The measurement of interrater agreement. In *Statistical Methods for Rates and Proportions*, 2nd edn, pp. 212–236. J. Wiley: New York

Heinen CD, Shivapurkar N, Tang Z, Groden J and Alabaster O (1996) Microsatellite instability in aberrant crypt foci from human colons. *Cancer Res* **56**: 5339–5341

Jass JR and Sobin LH (1993) Histological typing of intestinal tumors. In *WHO International Histological Classification of Tumours*, 2nd edn. Springer-Verlag: Berlin

Kinzler KW and Vogelstein B (1996) Lessons from hereditary colorectal cancer. *Cell* **87**: 159–170

Kouri M, Laasonen A, Mecklin J-P, Järvinen H, Franssila K and Pyrrhonen S (1990) Diploid predominance in hereditary nonpolyposis colorectal carcinoma evaluated by flow cytometry. *Cancer* **65**: 1825–1829

Leach FS, Nicolaides NC, Papadopoulos N, Liu B, Jen J, Parsons R, Peltomäki P, Sistonen P, Aaltonen LA, Nyström-Lahti M, Guan X-Y, Zhang J, Meltzer PS, Yu J-W, Kao F-T, Chen DJ, Cerosaletti KM, Fournier REK, Todd S, Lewis T, Leach RJ, Naylor SL, Weissenbach J, Mecklin J-P, Järvinen H, Petersen GM, Hamilton SR, Green J, Jass J, Watson P, Lynch HT, Trent JM, de la Chapelle A, Kinzler KW and Vogelstein B (1993) Mutations of a *mut*S homolog in hereditary non-polyposis colorectal cancer. *Cell* **75**: 1215–1225

Lee Y-S (1988) Background mucosal changes in colorectal carcinomas. *Cancer* **61**: 1563–1570

Losi L, Roncucci L, Di Gregorio C, Ponz de Leon M and Benhattar J (1996) K-*ras* and p53 mutations in human colorectal aberrant crypt foci. *J Pathol* **178**: 259–263

McKenzie KJ, Purnell DM and Shamsuddin AKM (1987) Expression of carcinoembryonic antigen, T-antigen and oncogene products as markers of neoplastic and preneoplastic colonic mucosa. *Hum Pathol* **18**: 1282–1286

McLellan EA and Bird RP (1988) Aberrant crypts: potential preneoplastic lesions in the murine colon. *Cancer Res* **48**: 6187–6192

Modica S, Roncucci L, Benatti P, Gafà L, Tamassia MG, Dardanoni L and Ponz de Leon M (1995) Familial aggregation of tumors and detection of hereditary non-polyposis colorectal cancer in 3-year experience of 2 population-based colorectal-cancer registries. *Int J Cancer* **62**: 685–690

Muto T, Bussey HJR and Morson BC (1975) The evolution of cancer of the colon and rectum. *Cancer* **36**: 2251–2270

Papadopoulos N, Nicolaides NC, Wei Y-F, Ruben SM, Carter KC, Rosen CA, Haseltine WA, Fleischmann RD, Fraser CM, Adams MD, Venter JC, Hamilton SR, Petersen GM, Watson P, Lynch HT, Peltomäki P, Mecklin J-P, de la Chapelle A, Kinzler KW and Vogelstein B (1994) Mutation of a *mut*L homolog in hereditary colon cancer. *Science* **263**: 1625–1629

Ponz de Leon M, Sassatelli R, Scalmati A, Di Gregorio C, Fante R, Zanghieri G, Roncucci L, Sant M and Micheli A (1993) Descriptive epidemiology of colorectal cancer in Italy: the six-year experience of a specialized registry. *Eur J Cancer* **29A**: 367–371

Pretlow TP, Barrow BJ, Ashton WS, O'Riordan MA, Pretlow TG, Jurcisek JA and Stellato TA (1991) Aberrant crypts: putative preneoplastic foci in human colonic mucosa. *Cancer Res* **51**: 1564–1567

Pretlow TP, Brasitus TA, Fulton NC, Cheyer C and Kaplan EL (1993) K-*ras* mutations in putative preneoplastic lesions in human colon. *J Natl Cancer Inst* **85**: 2004–2007

Pretlow TP, Roukhadze EV, O'Riordan MA, Chan JC, Amini SB and Stellato TA (1994) Carcinoembryonic antigen in human colonic aberrant crypt foci. *Gastroenterology* **107**: 1719–1725

Roncucci L, Stamp D, Medline A, Cullen JB and Bruce WR (1991*a*) Identification and quantification of aberrant crypt foci and microadenomas in the human colon. *Hum Pathol* **22**: 287–294

Roncucci L, Medline A and Bruce WR (1991*b*) Classification of aberrant crypt foci and microadenomas in human colon. *Cancer Epidemiol Biomarkers Prev* **1**: 57–60

Roncucci L, Pedroni M, Fante R, Di Gregorio C and Ponz de Leon M (1993) Cell kinetic evaluation of human colonic aberrant crypts. *Cancer Res* **53**: 3726–3729

Smith AJ, Stern HS, Penner M, Hay K, Mitri A, Bapat BV and Gallinger S (1994) Somatic *APC* and K-*ras* codon 12 mutations in aberrant crypt foci from human colons. *Cancer Res* **54**: 5527–5530

Shpitz B, Hay K, Medline A, Bruce WR, Bull SB, Gallinger S and Stern HS (1996) Natural history of aberrant crypt foci. *Dis Colon Rectum* **39**: 763–767

Thibodeau SN, Bren G and Schaid D (1993) Microsatellite instability in cancer of the proximal colon. *Science* **260**: 816–819

Weisburger JH and Wynder EL (1987) Etiology of colorectal cancer with emphasis on mechanisms of action and prevention. In *Important Advances in Oncology*,

© Cancer Research Campaign 1998 

DeVita VT, Hellman S, Rosenberg SA (eds), pp. 197–220. J.B. Lippincott: Philadelphia

Yamashita N, Minamoto T, Ochiai A, Onda M and Esumi H (1995) Frequent and characteristic K-*ras* activation and absence of p53 protein accumulation in aberrant crypt foci of the colon. *Gastroenterology* **108**: 434–440

Zanetti R and Crosignani P (1992) *Il cancro in Italia. I dati di incidenza dei Registri Tumori 1983–1987*, pp. 364–387. Associazione Italiana per la Lotta contro i Tumori, Associazione Italiana di Epidemiologia: Torino

*© Cancer Research Campaign 1998*